# Dynapenia is associated with a higher risk of depressive symptoms among older adults

**DOI:** 10.3389/fpubh.2025.1533973

**Published:** 2025-02-27

**Authors:** Chih-Ching Chang, Yung Liao, Jiaren Chen, Ting-Fu Lai, Ming-Chun Hsueh, Jong-Hwan Park, Yen-Jung Chang

**Affiliations:** ^1^Department of Health Promotion and Health Education, College of Education, National Taiwan Normal University, Taipei, Taiwan; ^2^Graduate Institute of Sport, Leisure and Hospitality Management, National Taiwan Normal University, Taipei, Taiwan; ^3^Faculty of Sport Sciences, Waseda University, Tokorozawa, Japan; ^4^Department of Convergence Medicine, Pusan National University School of Medicine, Yangsan, Republic of Korea; ^5^Graduate Institute of Sport Pedagogy, University of Taipei, Taipei, Taiwan; ^6^Department of Clinical Bio-Convergence, Graduate School of Convergence in Biomedical Science, Pusan National University School of Medicine, Yangsan, Republic of Korea; ^7^Convergence Medical Institute of Technology, Pusan National University Hospital, Busan, Republic of Korea

**Keywords:** handgrip strength, muscle weakness, sarcopenia, mental health, aging

## Abstract

**Background:**

Depression affects the global burden of disability among older adults. Although dynapenia is related to disability and potential psychological health outcomes, its association with depressive symptoms remains uncertain. Accordingly, the objective of the current study was to investigate this association in older Taiwanese adults, applying the 2019 Asian Working Group for Sarcopenia (AWGS) classification of dynapenia.

**Methods:**

Our research utilized a cross-sectional design implemented from September 2020 to December 2021, enrolling older adults aged over 65 years through National Taiwan University Hospital. The participants underwent standard assessments, including handgrip dynamometry for muscle strength, bioelectrical impedance analysis for muscle mass, and a 6-meter walk test for physical performance, to confirm the classification of dynapenia. The 15-item Geriatric Depression Scale (GDS-15) served as the tool to evaluate whether participants were at risk of potential depressive symptoms. The correlation between dynapenia and the risk of geriatric depressive symptoms was assessed through unadjusted and adjusted binary logistic regression analyses.

**Results:**

In total, 197 older adults (mean age was 80.5 ± 7.0 years; 52.8% female; 17.3% at risk of depressive symptoms; 55.8% with dynapenia) were included. Regardless of the different models, dynapenia remained significantly and positively related to the risk of geriatric depressive symptoms (OR [odds ratio]: 2.67; 95% CI [confidence interval]: 1.01–7.05; *p* = 0.048) after adjusting for potential confounders.

**Conclusion:**

Our findings highlighted a significant association between dynapenia, as classified by the 2019 AWGS criteria, and a higher risk of depressive symptoms in older Taiwanese adults. Public health professionals and practitioners should screen individuals with dynapenia for depressive symptoms to facilitate the early detection of depression. Future research should investigate the complex physiological and psychological mechanisms underlying this association.

## 1 Introduction

Depression is acknowledged as the most widespread mental health disorder worldwide (1). Globally, 280 million people were estimated to have depressive disorder in 2019, with the highest prevalence rates observed among older individuals aged 50–69 years (5.8%) and 70 years and above (5.4%) (2). As a major contributor to global years lived with disability (3), depressive disorders constitute a significant burden, impairing healthy aging by reducing the functional capacity among older adults (4). Depression in older adults is particularly concerning as it is associated with increased frailty, cognitive decline, suicidal ideation, and mortality (5–7). Similar to worldwide trends, an estimated 18.9 to 23.7% of older adults exhibit geriatric depressive symptoms in Taiwan (5, 8, 9). This high prevalence of depressive symptoms highlights a critical public health challenge in aging populations, emphasizing the need for early detection and targeted interventions to mitigate the adverse effects of late-life depression. Furthermore, previous longitudinal research revealed that older Taiwanese adults with worsening physical disability status had an association with depressive symptoms (10). This underscores the critical interplay between physical function decline and mental health deterioration in aging populations. Therefore, the factors related to age-associated decline in muscle strength or physical function that could affect geriatric depressive symptoms need to be understood better to promote healthy aging through the early detection and prevention of depression.

Dynapenia, characterized as low muscle strength due to aging (11, 12), is increasingly recognized as a critical public health issue (13–15). In Taiwan, the prevalence of dynapenia among older adults is estimated to range between 28.6 and 31.3% (16, 17), highlighting a substantial proportion of the aging population at risk for adverse health outcomes. Multiple studies have highlighted the physiological consequences of dynapenia in older populations (18), including increased risk of the locomotive syndrome (19, 20), reduced physical ability (21), and mortality (22, 23). However, existing studies on the psychological outcomes of dynapenia have primarily focused on cognitive function (17, 24, 25), and less attention has been given to its potential impact on other mental health outcomes such as depression (26, 27). Due to muscle weakness, dynapenia may exacerbate psychological distress by impairing mobility and increasing dependency on Instrumental Activities of Daily Living (21), which are well-established risk factors for depression (28, 29). However, despite evidence linking low muscle strength with an increased risk of depression (30, 31), the relationship between dynapenia, particularly as classified by the 2019 Asian Working Group for Sarcopenia (AWGS), and depressive symptoms remains unclear owing to the scarcity of research. Therefore, given the significant personal and societal burden of late-life depression, along with the high prevalence of dynapenia and geriatric depressive symptoms among older adults in Taiwan, a rigorous investigation of this association is crucial for informing early detection and intervention strategies to prevent further muscle decline and associated mental health risks.

We aimed to assess the association between dynapenia and the risk of depressive symptoms among older Taiwanese adults. Our hypothesis was that older adults with dynapenia had higher odds of depressive symptoms.

## 2 Materials and methods

### 2.1 Research design and recruitment

The current study employed a cross-sectional research design and was implemented from September 2020 to December 2021. A total of 299 participants aged 65 years and above with independent mobility were recruited through convenience sampling. The recruitment process followed two pathways. One approach involved the screening of potential participants who had completed their annual routine health examinations at National Taiwan University Hospital (NTUH) while the other relied on outpatient physicians from the Department of Geriatrics and Gerontology (DGG) at NTNU to identify eligible individuals. After the initial screening, eligible individuals were invited to participate, with eight refusing to consent. We ensured that the rights of all willing participants were protected and obtained a signed informed consent document from all of them before proceeding.

All participants completed a series of self-administered questionnaires during face-to-face interviews to evaluate their health status, health behaviors, and sociodemographic information, and the 15-item Geriatric Depression Scale (GDS-15) was employed to measure their risk of depressive symptoms. Subsequently, they underwent standardized procedures to evaluate muscle mass, strength, and physical performance to confirm whether their condition met the criteria of dynapenia based on the 2019 AWGS. Finally, to ensure accurate measurement of moderate-to-vigorous physical activity (MVPA), we required participants to wear a triaxial accelerometer for a continuous period of 7 days. After completing all procedures, participants were provided with a US$7 convenience store gift voucher in appreciation of their involvement. Approval and monitoring of this study were conducted by the Research Ethics Committee of the National Taiwan University Hospital (REC number: 202008046RINC).

**Figure 1:**
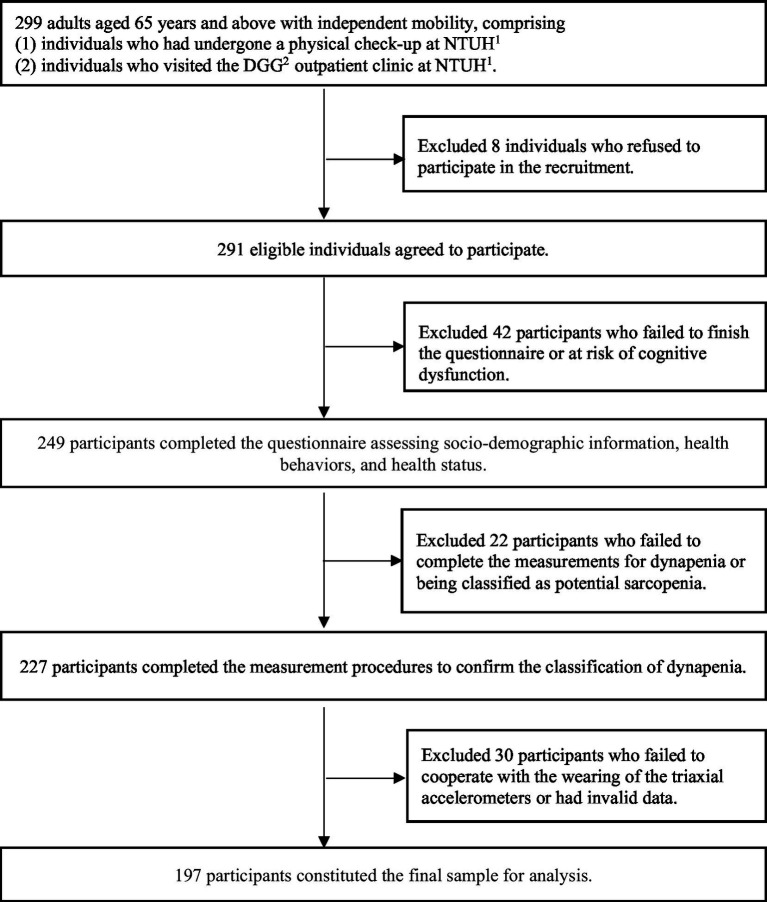
Process flow diagram for this study. NTUH^1^, National Taiwan University Hospital. DGG^2^, Department of Geriatrics and Gerontology.

In total, 291 individuals agreed to participate and were further screened based on the following exclusion criteria: (1) failure to complete the questionnaire or to be identified as at risk of cognitive dysfunction, as identified through Mini-Mental State Examination (MMSE) screening (*n* = 42); (2) inability to finish the standard procedures for assessing dynapenia or being classified as having potential sarcopenia (*n* = 22); and (3) uncooperative in wearing the triaxial accelerometer or the presence of invalid data (*n* = 30). The final analysis included 197 participants (Figure 1).

### 2.2 Measures

#### 2.2.1 Geriatric depressive symptoms

We utilized the GDS-15 scale to measure older adults’ risk of depressive symptoms (32, 33). The GDS-15 assessed the emotional state of participants, focusing on their experiences over the previous week. Responses of “yes” or “no” were recorded for each item. The range of GDS-15 scores was from 0 to 15, with a score of ≥5 suggesting participants at risk of depressive symptoms (34).

#### 2.2.2 Dynapenia

Dynapenia was defined as low muscle strength and/or poor physical performance with normal muscle mass (35, 36). Based on the 2019 AWGS criteria, standardized procedures were conducted, and the established cutoffs were applied to classify dynapenia by measuring participants’ muscle strength, physical performance, and muscle mass. Muscle strength was quantified using handgrip measurements with a hydraulic handheld dynamometer (Jamar Plus + Digital Hand Dynamometer, Lafayette Instrument Company, United States). Participants were directed to perform with maximum strength using their dominant hand while standing, and the process was repeated twice. A one-minute interval between the two attempts was allowed, and the attempt with the highest kilogram value was recorded. The highest handgrip strength data were recorded for analysis.

The walking speed of participants, measured via the 6-meter walk test, served as an assessment tool for physical performance. Each participant performed two walking trials, and the shortest duration was recorded for analysis. Professionals, adhering to standardized procedures, walked alongside the participants to ensure accuracy and recorded the time in seconds.

Bioelectrical impedance analysis was utilized in this study to quantify muscle mass. Participants were required to stand barefoot on a bioelectrical impedance analyzer (DC-430MA, TANITA, Tokyo, Japan) and maintain their grip on the measurement device with both hands until the measurement process was completed to ensure accurate readings.

Participants were categorized as having dynapenia if their handgrip strength was measured at <28.0 kg for men or <18.0 kg for women (indicating low muscle strength) or if their gait speed of <1.0 m/s for both sexes (indicating poor physical performance), while normal muscle mass, defined as ≥7.0 kg/m^2^ for men and ≥5.7 kg/m^2^ for women. To distinguish between the classifications of dynapenia and sarcopenia, participants with low muscle mass, potentially classifiable as sarcopenia (13), were excluded from the analysis to avoid overlap between these two conditions. Participants were classified as not having dynapenia if they exhibited normal muscle strength, physical performance, and muscle mass.

#### 2.2.3 Covariates

Sociodemographic data, such as age (categorized as 65 to 74 years or over 75 years), sex (female or male), and educational level (lower than university or university and above) was collected. Health behavior assessments focused on the patterns of substance use, specifically smoking and alcohol consumption. Alcohol consumption and smoking habits in the past year were categorized as “yes” or “no.” In health status assessments, chronic diseases were classified as fewer than 4 or 4 or more based on diagnoses by a physician. The calculation of body mass index (BMI) involved dividing body weight (kg) by height squared (m^2^). The Mini Nutritional Assessment Short-Form (MNA-SF) was employed to screen for nutritional risk among participants, with classifications of “at risk” for MNA-SF scores <12 (37–39). MVPA was objectively measured using a triaxial accelerometer (GT3X+ ActiGraph, Pensacola, Florida, United States), which participants wore for a week to precisely evaluate the average minutes per week of MVPA (40). In accordance with the WHO recommendation, the cutoff point for MVPA was set at a minimum of 150 min per week for older adults (41).

Previous studies have indicated that being a woman (42, 43), older age (44, 45), low educational attainment (46, 47), and BMI categories such as underweight (BMI < 18.5 kg/m^2^) (48), overweight (25 kg/m^2^ ≤ BMI < 30 kg/m^2^), and obesity (BMI ≥ 30 kg/m^2^) (49), as well as a high number of chronic diseases (50–52), tobacco use (current use of tobacco products), and alcohol consumption (alcohol consumption ≥1 time per week and ≥2 drinks per time) (53, 54) are associated with a higher risk of depressive symptoms in older populations. Furthermore, a relationship has been observed between the risk of nutrition (MNA-SF scores <12), evaluated using the MNA-SF, and depressive symptoms (55, 56). Moreover, older adults achieving ≥150 min of MVPA weekly demonstrated lower depressive symptoms (57). Based on these findings, the covariates included for analysis in this study were age, sex, educational level, BMI, smoking, alcohol drinking, chronic diseases status, nutritional status, and MVPA, all of which were adjusted in the logistic regression models.

### 2.3 Statistical analysis

Statistical analyses were conducted using IBM SPSS version 23.0 (SPSS Inc., Chicago, IL, United States). To summarize participant characteristics, descriptive statistics (frequency, percentage, and mean) were calculated, encompassing sociodemographic (age, sex, and educational level), health behaviors (smoking, alcohol drinking, and MVPA), and health status (BMI, chronic diseases, nutritional status, depressive symptoms, and dynapenia classified based on AWGS criteria). Chi-square (χ^2^) tests were utilized to analyze the correlations between categorical covariates and depressive symptoms. Analyses were performed using adjusted and unadjusted binary logistic regression models to examine the association between dynapenia and the risk of geriatric depressive symptoms. The independent variable was dynapenia (presence or absence), while the dependent variable was depressive symptoms (presence or absence). Three models were constructed to assess this association: Model 1 was an unadjusted model used to evaluate the crude association; Model 2 was adjusted for age and sex; and Model 3 was further adjusted, in addition to age and sex, for additional potential covariates, including educational level, BMI, smoking, alcohol consumption, chronic disease status, nutritional status, and MVPA. The results were described in the form of odds ratios (OR) and 95% confidence intervals (CI). A threshold of *p* < 0.05 was employed to define the statistical significance.

## 3 Results

In total, 197 participants were analyzed, with Table 1 presenting their sociodemographic, health behavior, and health status variables. Participants included 52.8% women, with the mean age of 80.5 ± 7.0 years. Approximately half had university-level education or higher (50.8%), and a normal BMI (50.3%). The majority of participants did not use tobacco (92.9%) or alcohol (90.9%). 32.0% of participants achieved at least 150 min/week of MVPA. Most participants had normal nutritional status (80.2%), and fewer than four chronic diseases (87.8%). A total of 110 (55.8%) participants had dynapenia based on the 2019 AWGS definition, whereas 34 (17.3%) were identified as being at risk for depressive symptoms. Significant associations between educational level, smoking status, nutritional status, and dynapenia with the risk of geriatric depressive symptoms were identified in the chi-square test. The group at risk of depressive symptoms had a higher proportion of participants with dynapenia.

**Table 1:** Study participant demographics and characteristics.

Variables	Total (*n* = 197) N (%)	Risk of depressive symptoms		*p*-value
		No (*n* = 163) N (%)	Yes (*n* = 34) N (%)	
Age				
65–74	50 (25.4%)	37 (22.7%)	13 (38.2%)	0.058
75 and over	147 (74.6%)	126 (77.3%)	21 (61.8%)	
Sex				
Female	104 (52.8%)	86 (52.8%)	18 (52.9%)	0.985
Male	93 (47.2%)	77 (47.2%)	16 (47.1%)	
Educational level				
Lower than university	97 (49.2%)	75 (46.0%)	22 (64.7%)	0.047^*^
University and over	100 (50.8%)	88 (54.0%)	12 (35.3%)	
Smoking				
No	183 (92.9%)	155 (95.1%)	28 (82.4%)	0.018^*^
Yes	14 (7.1%)	8 (4.9%)	6 (17.6%)	
Alcohol drinking				
No	179 (90.9%)	149 (91.4%)	30 (88.2%)	0.522
Yes	18 (9.1%)	14 (8.6%)	4 (11.8%)	
Chronic diseases				
Fewer than four	173 (87.8%)	143 (87.7%)	30 (88.2%)	0.935
Four or more	24 (12.2%)	20 (12.3%)	4 (11.8%)	
BMI				
Underweight	6 (3.0%)	5 (3.0%)	1 (2.9%)	0.638
Normal	99 (50.3%)	80 (49.1%)	19 (55.9%)	
Overweight	53 (26.9%)	43 (26.4%)	10 (29.4%)	
Obesity	39 (19.8%)	35 (21.5%)	4 (11.8%)	
Nutritional status				
Normal	158 (80.2%)	139 (85.3%)	19 (55.9%)	< 0.001^***^
At risk	39 (19.8%)	24 (14.7%)	15 (44.1%)	
MVPA				
< 150 min/per week	134 (68.0%)	108 (66.3%)	26 (76.5%)	0.245
≥ 150 min/per week	63 (32.0%)	55 (33.7%)	8 (23.5%)	
Dynapenia				
No	87 (44.2%)	79 (48.5%)	8 (23.5%)	0.008^**^
Yes	110 (55.8%)	84 (51.5%)	26 (76.5%)	

BMI, Body mass index; MVPA, Moderate-to-vigorous physical activity. ^*^p < 0.05; ^**^p < 0.01; ^***^p < 0.001.

In Table 2, three different logistic regression models present the assocaitaion between dynapenia and the odds of depressive symptoms. Model 1 represented the unadjusted analysis. Model 2 accounted for age and sex, while model 3 included adjustments for age, sex, educational level, BMI, chronic disease status, smoking, alcohol consumption, nutritional status, and MVPA. Dynapenia demonstrated a significant association with the risk of geriatric depressive symptoms across model 1 (OR: 3.06; 95% CI: 1.31–7.15; *p* = 0.010), model 2 (OR: 3.12; 95% CI: 1.32–7.36; *p* = 0.009), and model 3 (OR: 2.67; 95% CI: 1.01–7.05; *p* = 0.048). Notably, nutritional risk (OR: 4.60; 95% CI: 1.77–11.95; *p* = 0.002) was also significantly associated with the risk of geriatric depressive symptoms in model 3.

**Table 2:** Logistic regression analysis of the association of dynapenia with depressive symptoms (*n* = 197).

	Model 1		Model 2		Model 3	
	OR (95%CI)	*p*-value	OR (95%CI)	*p*-value	OR (95%CI)	*p*-value
Dynapenia						
No	1.00		1.00		1.00	
Yes	3.06(1.31, 7.15)	0.010*	3.12 (1.32, 7.36)	0.009**	2.67 (1.01, 7.05)	0.048*
Age						
65–74			1.00		1.00	0.138
≥ 75			0.46 (0.21, 1.03)	0.059	0.50 (0.20, 1.25)	
Sex						
Female			1.00		1.00	0.637
Male			1.01 (0.47, 2.17)	0.976	1.26 (0.48, 3.36)	
Educational level						
Lower than university					1.00	0.259
University and over					0.59 (0.23, 1.48)	
Smoking						
No					1.00	0.104
Yes					3.64 (0.77, 17.31)	
Alcohol drinking						
No					1.00	0.383
Yes					0.47 (0.08, 2.58)	
Chronic diseases						
Fewer than four					1.00	0.771
Four or more					0.81 (0.20, 3.31)	
BMI						
Underweight					1.00	0.323
Normal					2.19 (0.19, 25.73)	
Overweight					2.29 (0.18, 28.92)	
Obesity					0.65 (0.04, 9.95)	
Nutritional status						
Normal					1.00	0.002**
At risk					4.60 (1.77, 11.95)	
MVPA						
< 150 min/per week					1.00	0.623
≥ 150 min/per week					0.78 (0.29, 2.12)	

BMI, Body mass index; MVPA, Moderate-to-vigorous physical activity. Model 1: No adjustments; Model 2: Adjusted for age and sex; Model 3: Adjusted for age, sex, educational level, BMI, chronic disease status, smoking, alcohol consumption, nutritional status, and MVPA. ^*^p < 0.05; ^**^p < 0.01.

## 4 Discussion

Based on the available evidence, the current study was the first to explore dynapenia, classified strictly by the 2019 AWGS criteria, and its association with the risk of depressive symptoms among older adults in Taiwan. Our main finding revealed a significant positive association between dynapenia and higher risk of geriatric depressive symptoms, following adjustments for several potential confounders. These results could be valuable for public health professionals and practitioners, suggesting strategies for the early screening and prevention of depressive symptoms in the older population.

Consistent with prior studies, this research highlights a connection between dynapenia and depressed mood among older adults (26, 27) and further reports that, even when the more rigorous 2019 AWGS criteria were applied, dynapenia remained related to a higher risk of geriatric depressive symptoms in an Asian population. The Irish study demonstrated a significant association between dynapenia and higher odds of both incident and persistent depressive symptoms among community-dwelling older adults aged over 50 years (26). Similarly, the Korean study reported that dynapenia was associated with an increased risk of depressive symptoms among older adults, particularly in men (27). Our results are in line with these international research and emphasize the community-dwelling older adult population. As the aging population continues to expand, community-dwelling older adults represent a broader at-risk group, highlighting the public health importance of identifying and managing dynapenia as a modifiable risk factor for geriatric depressive symptoms.

There are several possible underlying explanations for the observed relationship between dynapenia and depressive symptoms. First, dynapenia, defined as age-related muscle weakness, substantially impairs older adults’ physical ability (11, 15) to be involved in fundamental and instrumental tasks of daily living (21, 58), leading to a decreased sense of independence and increased frustration, possibly contributing to the onset and exacerbation of depressive symptoms (28, 59). Second, dynapenia is associated with age-associated physiological changes, including a reduction in motor cortex neurotrophic factors and diminished neurotransmitter levels, such as dopamine and serotonin (15), both of which could lead to the worsening of depressive symptoms (60, 61). Third, beyond physical impairment, older adults with dynapenia are at an increased risk of social challenges. Limitations in mobility and daily activities restrict their ability to engage in social interactions, reducing social participation and interpersonal communication. This heightens loneliness and the experience of social isolation (62) and may ultimately increase depressive symptoms with age (63, 64). Since dynapenia may be related to geriatric depressive symptoms through physical, physiological, and social pathways, understanding the relationship between dynapenia and depression would be important. Future studies should explore the mechanisms linking dynapenia to depression.

Furthermore, in addition to dynapenia, we also observed a significant association between nutritional risk and geriatric depressive symptoms, which is consistent with previous studies (55, 56). Moreover, a randomized controlled trial demonstrated that a combined resistance training and diet intervention significantly improved depressive symptoms among older adults (65). These findings further emphasize the need for targeted physical activity and nutritional interventions to mitigate the risk of late-life depression in older populations.

This study was the first to investigate the relationship between dynapenia and the risk of depressive symptoms among the older population by strictly adhering to the 2019 AWGS standard criteria in Taiwan. Previous studies have demonstrated that weaker handgrip strength (66–68) and slower gait speed (69, 70) are associated with depressive symptoms. However, variability in the diagnostic standards employed across these studies might affect the comparability and interpretation of the findings. Hence, we used the definition of dynapenia, according to the AWGS 2019 guidelines, to align our methodologies with the global research standards. In addition to being specifically tailored to Asian populations, this approach ensured that our findings are robust, internationally comparable, and applicable to geriatric health.

This study has some limitations should be acknowledged. First, the cross-sectional research design restricted the ability to establish causality. It remains unclear whether dynapenia contributes to the development of depressive symptoms or if depressive symptoms lead to reduced physical activity, resulting in muscle weakness. Therefore, longitudinal studies are needed to clarify the directionality of this relationship and investigate the underlying mechanisms. Second, participation in routine health checkups was voluntary, which may have resulted in a selection bias toward individuals who were more attentive to their health. Additionally, the recruitment of participants from a single medical center limits the generalizability of the findings to broader aging populations. Future research should aim to incorporate more diverse sample from various community settings and healthcare institutions, to enhance the external validity of the findings. Third, high scores on the GDS-15 do not necessarily imply a diagnosis of clinical depression; therefore, interpretation of the results should consider diagnostic limitations. Future research should incorporate clinical diagnostic assessments to enhance validity. Finally, some potential confounders, such as chronic inflammation, stress (71–73), and sleep disturbances (74–76), commonly associated with aging, may contribute to the relationship between dynapenia and depression. Future studies should account for these confounders to ensure more accurate interpretation.

Despite these limitations, the findings of this study have important public health implications for addressing the intersection of muscle strength decline and mental health in aging populations. Given the increasing prevalence of both dynapenia and geriatric depressive symptoms, early detection and intervention strategies are essential to mitigate the burden of late-life depression. Public health initiatives should integrate muscle function screening into routine geriatric health assessments, particularly in community settings, to identify individuals with dynapenia before they develop severe physical or psychological consequences. Community-based interventions promoting physical activity and nutrition status should be emphasized to prevent the risk of depressive symptoms in older adults.

## 5 Conclusion

Dynapenia demonstrated a significant relationship with a higher risk of depressive symptoms in older Taiwanese adults. Public health professionals and practitioners should screen older individuals with reduced muscle strength or poor physical performance for depressive symptoms to enable early detection of their depression. Further research should investigate the physiological and psychological pathways through which dynapenia influences geriatric depressive symptoms.

## Data Availability

The raw data supporting the conclusions of this article will be made available by the authors, without undue reservation.
